# Objective color calibration for manufacturing facial prostheses

**DOI:** 10.1117/1.JBO.26.2.025002

**Published:** 2021-02-13

**Authors:** Yargo V. Tessaro, Sérgio S. Furuie, Denise M. Nakamura

**Affiliations:** a University of São Paulo, School of Engineering, Biomedical Engineering Laboratory, São Paulo, Brazil; b University of São Paulo, School of Dentistry, Department of Surgery, Maxillofacial Prosthesis, and Traumatology, São Paulo, Brazil

**Keywords:** maxillofacial prosthesis, skin imaging, color correction, image processing, image calibration

## Abstract

**Significance:** Rehabilitation through facial prostheses’ main goal is to aid individual’s social reintegration as well as improving their quality of life. However, this treatment is not yet widely available in Brazil due to the lack of specialized clinics and the cost associated with the high number of necessary medical appointments until the final result. One of the steps in the process consists of measuring skin color, which is observer-dependent and may suffer from the effect of metamerism.

**Aim:** The methodology of our work aims to obtain a standard between different devices and greater fidelity to the color seen in person in order to reduce face-to-face iterations, reduce costs, and ensure better final results.

**Approach:** A physical device and a computer program were improved from previous projects. The changes included implementing the Thin-Plate Spline 3D algorithm for color calibration, in addition to an optional non-uniform illumination correction in the process. We also aim to improve the project’s accessibility using a colorimeter. The methodology and the algorithms were both compared to readings from direct skin measurements as well as color references.

**Results:** After processing, the $\Delta E_{ab}^{*}$ metric between images from the same segments is taken with different cameras and conditions of illumination decreased from 18.81±4.85 to 4.85±1.72. In addition, when the images were compared to colorimetric readings of the skin, the difference went from 14.93±4.11 to 5.85±1.61. It was also observed that using a less expensive device did not impact the readings. The project is open source and available at Github.

**Conclusions:** The results demonstrate the possibility of applying the methodology to assist in the manufacturing of facial prostheses to decrease the total number of consultations, in addition to providing greater reliability of the final result.

## 1 Introduction

Losses and malformations of the face, such as the loss of the ear, cause morphofunctional changes and, consequently, several psychosocial effects, such as depression, sadness, shame, anxiety, and anger.^1^^,^^2^ Thus in some cases, prosthetic facial rehabilitation can be established.

**Fig. 1:**
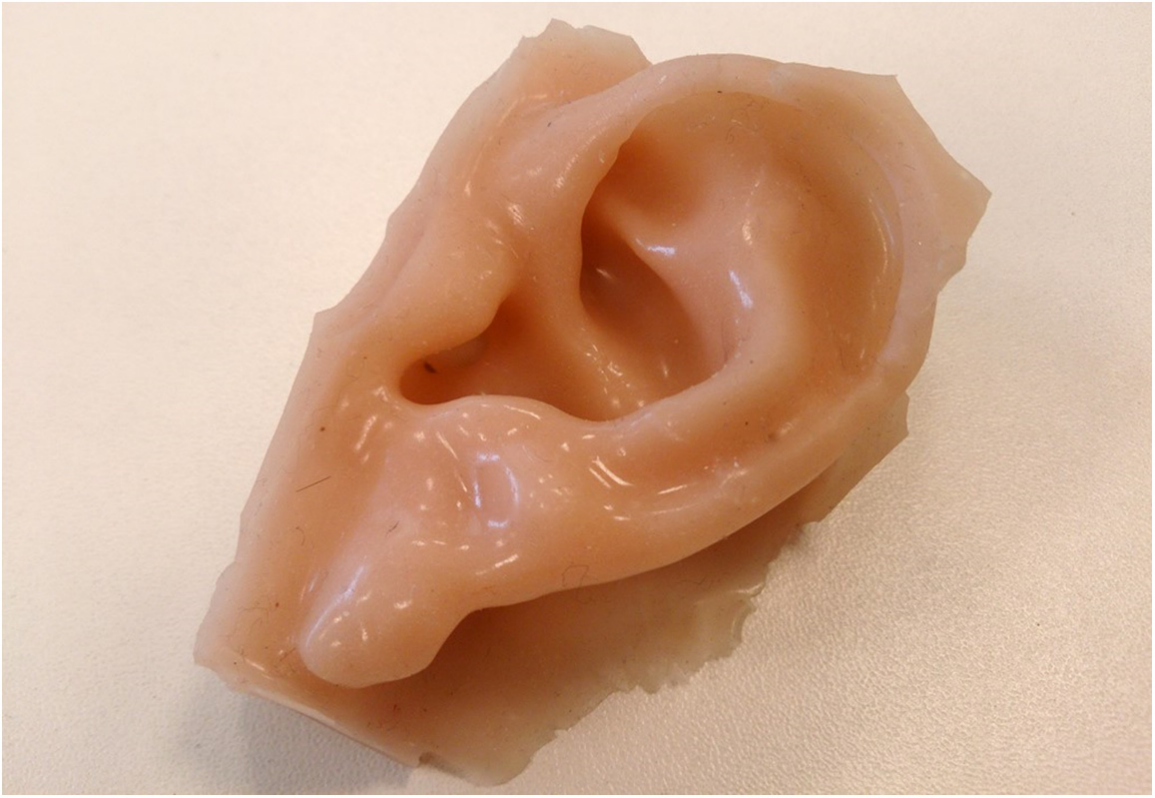
Example of facial prosthesis (courtesy of the Maxillo Facial Prosthesis clinic at the School of Dentistry of University of São Paulo).

Alloplastic facial prostheses’ (Fig. 1) goal is to provide anatomical, functional, and aesthetic rehabilitation of facial losses and malformations^3^ helping in the social reintegration of the individual and, consequently, improving their quality of life.^1^^,^^2^^,^^4^^–^^6^ Even today, some factors, such as the scarcity of specialized clinics, the time, and money spent associated with the number of necessary consultations to obtain the final result and the large extension of the national territory, make it difficult for most patients to receive treatment in Brazil.

In order to decrease the number of consultations for each patient and, consequently, the time necessary for making facial prostheses, several studies propose the use of technologies.^7^^–^^14^ However, presential steps are still needed to carry out the anamnesis, impression or scanning of the defect, check the marginal adaptation, extrinsic coloration, and delivery of the final prosthesis.

In this process, a fundamental step is measuring the patient’s skin color. However, this determination is also observer-dependent, which may suffer from the effects of metamerism, characterized by the inability to distinguish differences in the color spectrum of objects. This is caused because, under different illuminations, the human eye’s trichromatic response can define similar colors.^15^^,^^16^ For this, there are commercial spectrophotometers capable of obtaining the color profile in an analytical way, already adapting it to the pigments to be used. However, they have a relatively high cost and work with imported pigments, making it difficult to be used especially in the public sector.

A possible low-cost solution to reduce subjectivity in the process of color measurement and repetition would be the use of calibration and restoration processing of images obtained by mobile devices. This method is already used in the area of medicine called teledermatology, in which the diagnosis is made through the remote analysis of the patient’s skin images since the fidelity of the color presented to the health professional is a fundamental factor in the diagnosis of skin cancer. Similarities between diagnostics using remote images after processing and face-to-face consultation with correlation close to 100% are found in the literature.^17^^–^^23^

**Fig 2:**
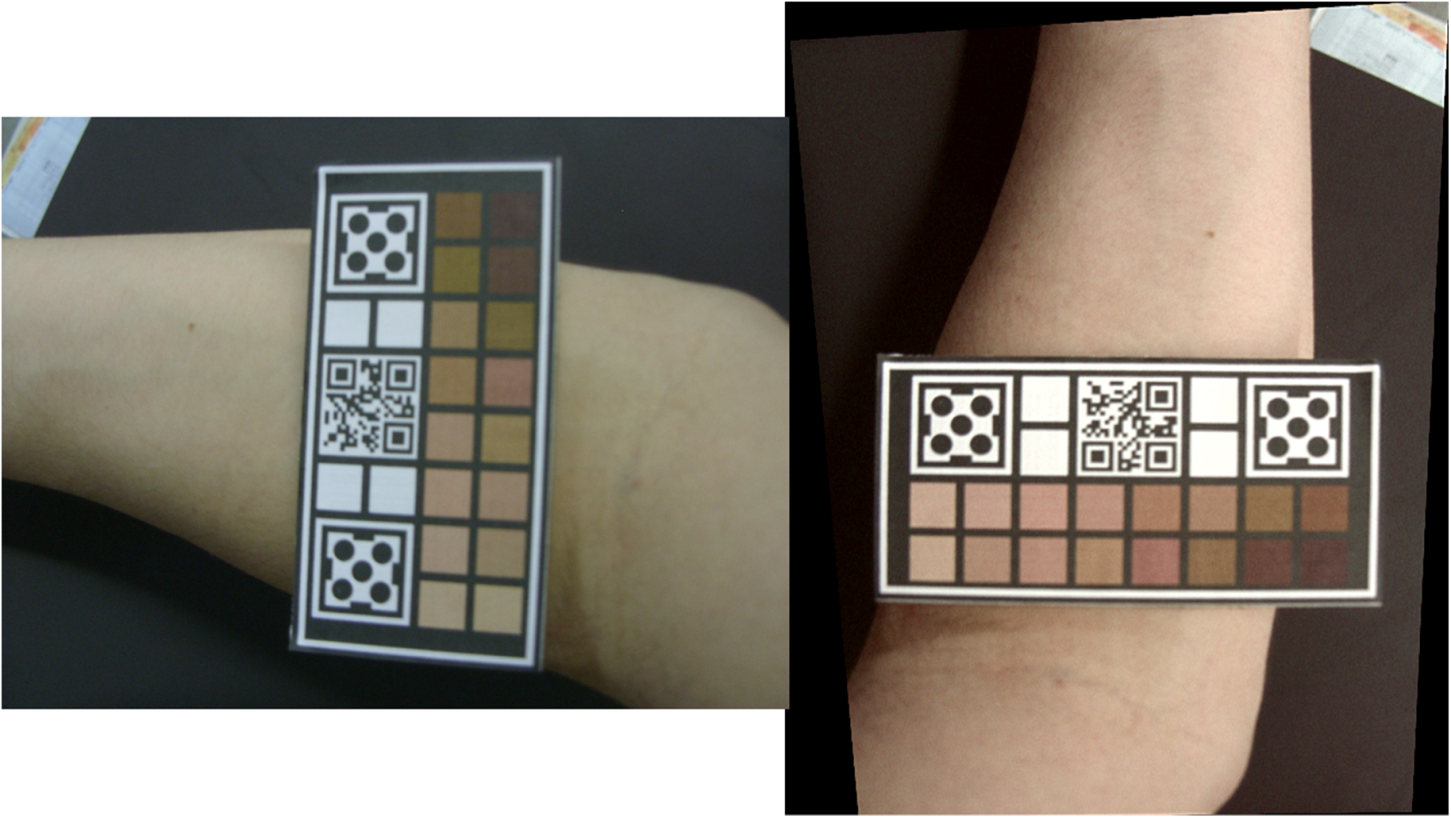
Demonstrative example of the previously developed methodology, which corrected color, perspective, and resolution of the image.

Among the challenges encountered in image processing, the existence of different color standards between devices and effects caused by illumination or degradation of the quality of the photograph can be highlighted. In a previous work,^24^ a methodology was proposed to calibrate and restore images in teledermatology in order to develop a system composed of a physical device (Fig. 2) and software capable of calibrating and evaluating images of skin taken from conventional and phone cameras, enabling a standard between different imaging devices. In this way, this project consists of adapting and improving the developed process focusing on the area of maxillofacial prostheses.

After assessing colors, the correct formulation is necessary so that the pigments used in the manufacturing of the prosthesis are consistent with the adjacent tissues.^25^^,^^26^ This work is part of a study that aims to generate a formulation of colors in the manufacture of facial prostheses from the obtained images.

## 2 Methods

### 2.1 Color Correction

The first fundamental point when dealing with color images is the fact that each device has a different representation of the RGB color system. In this way, images of the same objects taken with cameras or presented by different printers can cause different visual perceptions for an observer.

In order to distinguish such effects, the CIELAB (LAB) color space is proposed, which is obtained through spectrometric color readings and can be converted to the sRGB space, comparable to the commonly used RGB.^27^^–^^29^ Within the LAB space, the $\Delta E_{ab}^{*}$ metric, defined as the Euclidean distance between two LAB vectors, is generally used to compare color perception and the lower the result, the more difficult it is for an observer to distinguish between two colors, with 3.00 being a threshold value accepted for human differentiation.^20^^,^^25^^,^^30^

The algorithm used previously, based on the definition of a weight matrix $A_{\left\{3,4\right\}}$ that minimizes the mean square error between a set of reference values and the set of measured values, provides good results and captures the linear variations of the image.^31^ However, it is not able to determine non-linear corrections for the images, being one of its limitations.

**Fig. 3:**
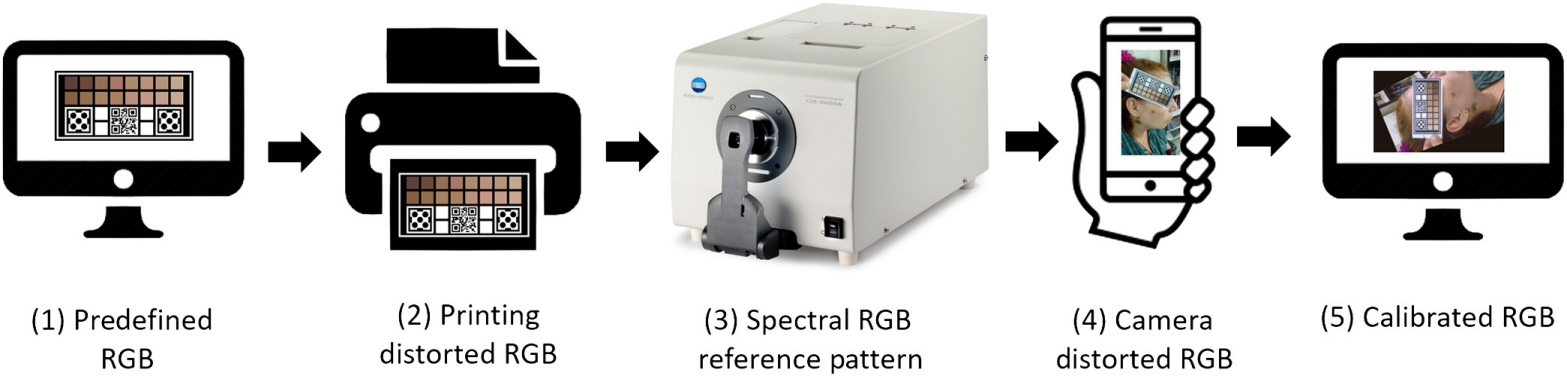
Diagram of the main steps and different RGB values associated with the evaluation of correction algorithm.

In this calibration process, it is important to highlight the different RGB values present in devices, indicating the reference used in the piece of software. Figure 3 shows the different steps in the calibration process with 16 tonalities. In this work, a spectrophotometer (CM-3600A, Konica Minolta, Japan) was used.

For this, the use of an algorithm capable of correcting both linear and non-linear transformations was proposed. This algorithm, called “3D Thin-Plate Spline” (TPS-3D) in the literature, corresponds to a mapping between points in different spaces in order to interpolate them minimizing the bending energy, defined by the integral of the sum of squared second derivatives.^32^ Since this mapping is done by a spline of a non-linear function, the algorithm is able to determine corrections that a simple matrix transformation would not.

In this algorithm, matrices W and A are calculated by

$$[\begin{array}{c} W\\ A \end{array}]={\left[\begin{array}{cc} K & P\\ P^{T} & 0_{\left\{4,4\right\}} \end{array}\right]}^{-1}[\begin{array}{c} V\\ 0_{\left\{4,3\right\}} \end{array}],$$ (1)

in which the matrices $0_{\left\{4,4\right\}}\;e\;0_{\left\{4,3\right\}}$ are zeros matrices with dimensions 4×4 and 4×3; P (and its transpose $P^{T}$) is the matrix of the average RGB points of each of the 16 segments read (corresponding to step 4 of Fig. 3) with the addition of a 1-in. each line, indicated by

$$P=[\begin{array}{cccc} 1 & R_{1} & G_{1} & B_{1}\\ 1 & R_{2} & G_{2} & B_{2}\\ \ldots & \ldots & \ldots & \ldots \\ 1 & R_{16} & G_{16} & B_{16} \end{array}].$$ (2)

K is a matrix of U(r) for shape distortion by TPS-3D, defined by

$$K=[\begin{array}{ccccc} 0 & U(r_{12}) & \ldots & U(r_{1,15}) & U(r_{1,16})\\ U(r_{2,1}) & 0 & \ldots & U(r_{2,15}) & U(r_{2,16})\\ \ldots & \ldots & \ldots & \ldots & \ldots \\ U(r_{15,1}) & U(r_{15,2}) & \ldots & 0 & U(r_{15,16})\\ U(r_{16,1}) & U(r_{16,2}) & \ldots & U(r_{16,15}) & 0 \end{array}],\;U(r_{i,j})=2(r_{i,j}^{2})\log(r_{i,j}+10^{-20}),$$ (3)

where

$$r_{i,j}=\sqrt{(R_{i}-R_{j}{)}^{2}+(G_{i}-G_{j}{)}^{2}+{\left(B_{i}-B_{j}\right)}^{2}}.$$ (4)

Finally, V is the matrix of RGB color references of the 16 points (corresponding to step 3 of Fig. 3), given by

$$V=[\begin{array}{ccc} R_{1}^{\prime } & G_{1}^{\prime } & B_{1}^{\prime }\\ \cdots & \cdots & \cdots \\ R_{16}^{\prime } & G_{16}^{\prime } & B_{16}^{\prime } \end{array}]$$ (5)

In this model, matrices W and A are used to correct a pixel with values $R_{\mathrm{meas}},G_{\mathrm{meas}},B_{\mathrm{meas}}$ using the formulation given by

$$[\begin{array}{ccc} R_{\mathrm{calib}} & G_{\mathrm{calib}} & B_{\mathrm{calib}} \end{array}]=[\begin{array}{cc} K_{\mathrm{meas}} & P_{\mathrm{meas}} \end{array}][\begin{array}{c} W\\ A \end{array}],$$ (6)

with $K_{\mathrm{meas}}$ and $P_{\mathrm{meas}}$ defined as

$$P_{\mathrm{meas}}=[\begin{array}{cccc} 1 & R_{\mathrm{meas}} & B_{\mathrm{meas}} & G_{\mathrm{meas}} \end{array}],$$ (7)

$$K_{\mathrm{meas}}=[\begin{array}{cccc} U_{\mathrm{meas}}(r_{1}) & U_{\mathrm{meas}}(r_{2}) & \ldots & U_{\mathrm{meas}}(r_{16}) \end{array}],$$ (8)

in which the function $U_{\mathrm{meas}}(r_{i})$ and $r_{i}$ are given by

$$U_{\mathrm{meas}}(r_{i})=2(r_{i}^{2})\log(r_{i}+10^{-20}\;)$$ (9)

$$r_{i}=\sqrt{(R_{\mathrm{meas}}-R_{i}{)}^{2}+(G_{\mathrm{meas}}-G_{i}{)}^{2}+(B_{\mathrm{meas}}-B_{i}{)}^{2}}.$$ (10)

It is important to emphasize that the method of this work is used to approximate skin colors since they are closer to the reference model, thus reducing the expected error within the interpolated space. Likewise, colors distant from this standard can present greater relative errors.

**Fig. 4:**
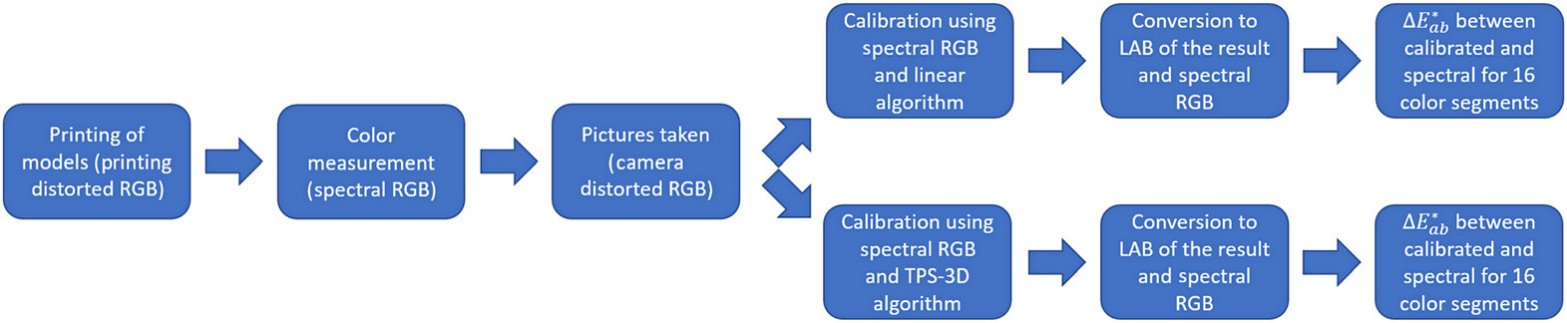
Flowchart for algorithm comparison with steps of image acquisition and reference values used for both cases.

In order to verify the improvement of the results from the change in algorithm, both were used in the same set of images obtained by printing the models on two different printers (Officejet Pro 8600 and Deskjet 2546, Hewlett-Packard, United States) and photographs taken by two cell phones [Moto G4, Motorola, United States; Pixi 4 (3.5ʺ), Alcatel, France] with different camera resolutions (13 and 5 MP), following the flowchart of Fig. 4.

### 2.2 Illumination Correction

Then an attempt was made to obtain an illumination correction so as not to depend on uniform lighting to carry out the calibration process and, thus, obtain more reliable and robust results.

This process is commonly called background subtraction and consists of using a characteristic representation of the background of the image to perform the correction.^33^^,^^34^

In practice, it is necessary to estimate the image with illumination I(x,y) so that the following operation can be performed:

$$G(x,y)=\frac{F(x,y)}{I(x,y)}\cdot C,$$ (11)

in which G(x,y) is the illumination corrected image, F(x,y) is the original image, and C is a constant so that the luminosity in G is consistent with F, defined by:$C=\mathrm{mean}(F(x,y))\cdot \frac{1}{\mathrm{mean}(\frac{F(x,y)}{I(x,y)})}$

First, it is necessary to point out that this correction must be made only in the L channel of the image’s LAB space—corresponding to the luminosity—and, if it was made in the other channels or using the RGB space, the color result could be changed unpredictably.

In this work, the estimation of I(x,y) was done using a Gaussian filter with standard deviation (σ) equal to half of the largest dimension of the whole image, being a low-pass filter responsible for maintaining global image characteristics.

The project with both changes was made available in full at https://github.com/yargo13/correct_image and is developed as a plugin for the ImageJ platform, however, it has not yet been migrated to version 2 of the framework.^35^

### 2.3 Project Accessibility

In order to increase the accessibility of the project, one of the main points is to use a lower cost alternative to obtain the spectral values of colors in the printed device, which corresponds to step 3 of Fig. 3. For this, the use of a hand-held colorimeter (ColorMunki, X-Rite, Grand Rapids, United States) was verified.

The device consists of a spectrophotometer for use with imaging devices such as projectors and printers. Therefore, it has a lower cost when compared to devices that have a scientific purpose with a spectrum of broader range, not restricted to visible light. In addition, it is also more portable due to its dimensions.

**Fig. 5:**
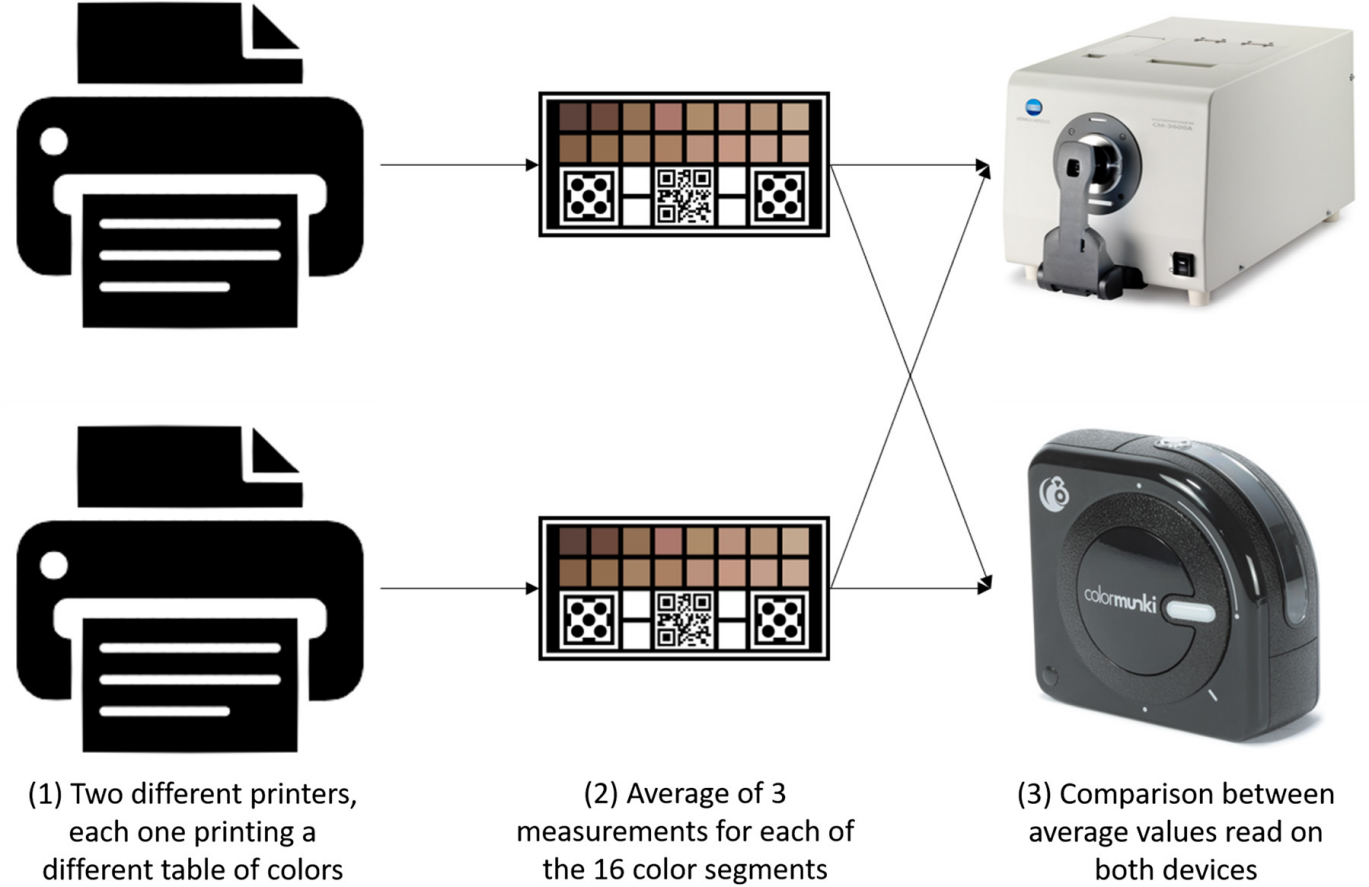
Diagram of device comparison analysis.

The objective of this step was to verify the reproducibility of the readings made previously with the CM-3600A spectrophotometer in comparison to the ColorMunki Photo, both before calibrations. For this, using two devices printed by different printers, three readings of the LAB values of each color segment were made using the ColorMunki Photo and averaged afterward, then, they were compared to the measurements made with the spectrophotometer. A diagram regarding these steps is presented in Fig. 5. In this comparison, it is important to verify the illuminant settings used since the spectrophotometer showed readings for the D65 illuminant while the X-Rite product used the D50 illuminant. Therefore, for an effective comparison, the reflectance values read on the spectrophotometer were converted to LAB using the D50 illuminant. This conversion is established in the literature.^36^

### 2.4 Temporal Degradation

The first step to study the temporal deterioration of the model was to compare measurements made with a difference of 13 months using the same spectrophotometer. Based on this result, it would be possible to conclude if there were variations in the color spectrum, characteristic of the studied phenomenon.

In this comparison, the LAB values of all segments were read for three devices obtained from two printers, totaling six devices. The data obtained were compared in two ways.

1. The values of similar segments were averaged in each device and the analysis is made considering 16 values for each printer.
2. The 96 values of each segment (16 for each of the six devices) were analyzed individually with the previous values.

**Fig. 6:**
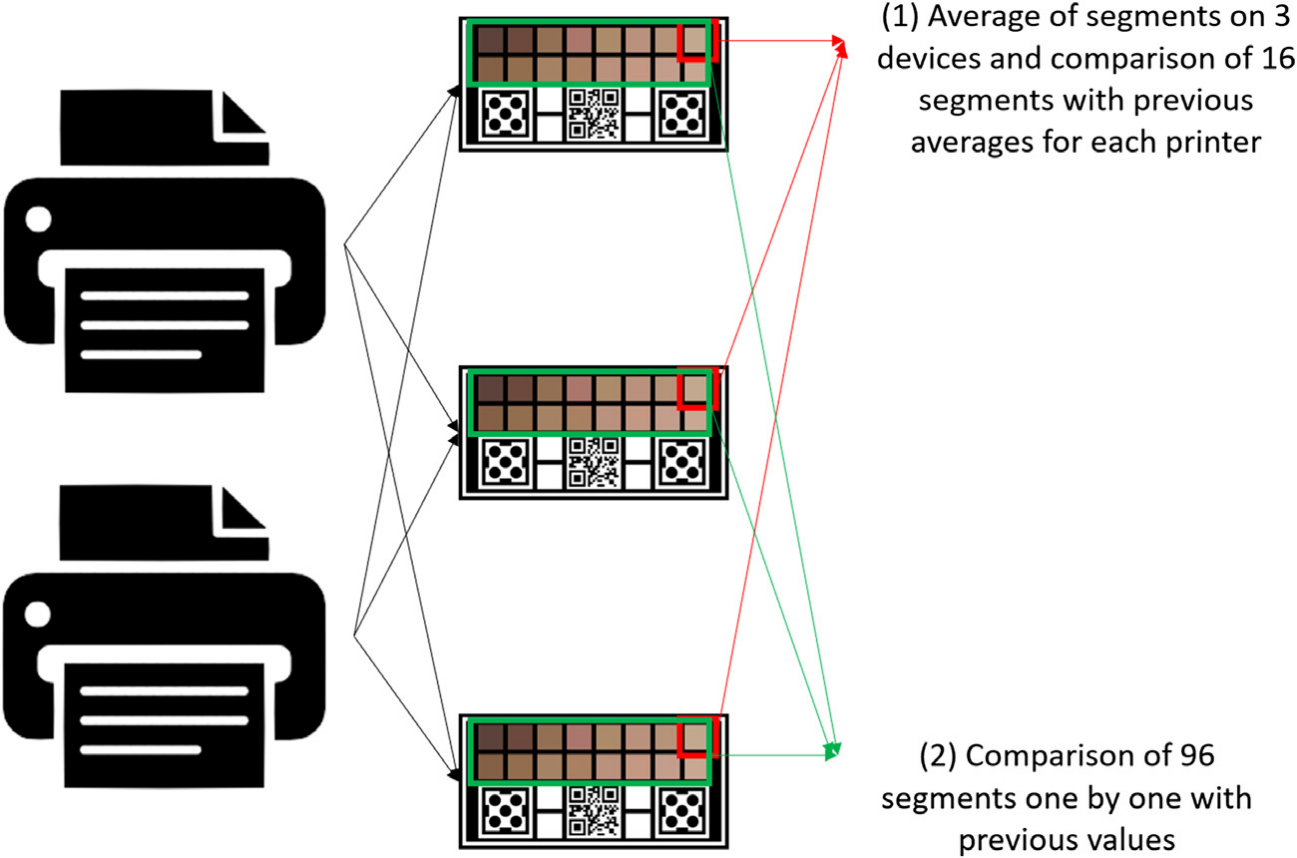
Diagram of temporal degradation analysis with spectrophotometer, specifying the two comparisons used.

A diagram representing this analysis can be seen in Fig. 6. In this analysis, in addition to comparing the values using the $\Delta E_{ab}^{*}$ metric, a statistical analysis was performed according to the Bland–Altman method.^37^

### 2.5 Skin Measurements

During the last stage of the project, the calibration methodology developed was evaluated through a procedure, in which the final values of the LAB colors in the image were compared with values read using the ColorMunki colorimeter. With that, the intention was to verify the applicability of the system and shows possible improvements for future projects.

**Fig. 7:**
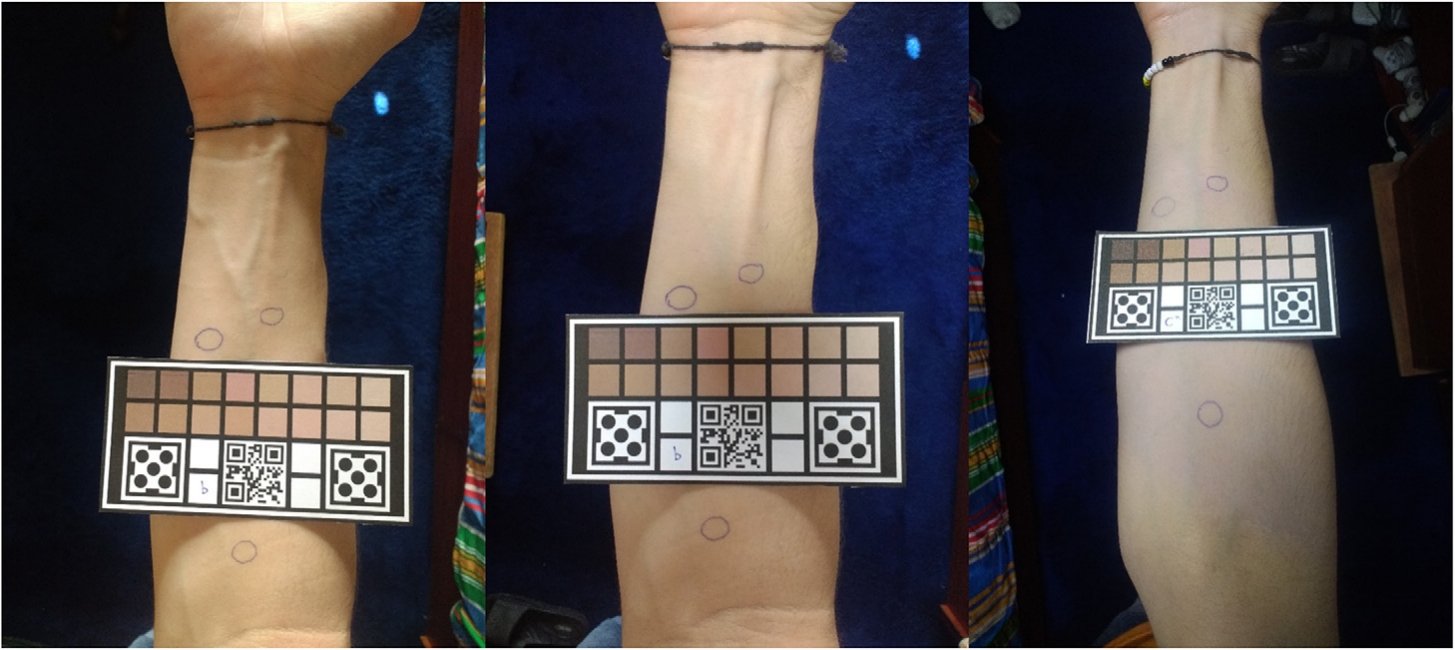
Comparison of the same skin segment in different illumination situations with regions of interest.

For this, photographs were taken containing skin segments using two cell phones and two calibration models under different illumination conditions. In each skin segment, regions of interest that were small enough to be specific, but that allowed a color measurement not affected by the marking, were manually established (Fig. 7).

Even though the purpose of the current project is to use the algorithm to match face tones, the pictures on this part were taken of the forearm due to ease of operation and based on the assumption that skin tone variability between different anatomical parts does not present significant differences.

Each region of interest had its LAB component value measured in three situations:

(1) using the colorimeter, with the average between three readings;
(2) using the piece of software to average the LAB results of the manually selected region before the calibration;
(3) using the piece of software to average the LAB results of the manually selected region after the calibration.

For this study, measurement 1 will be adopted as a reference for a human observer. However, there is evidence in the literature that measurements using contact-based equipment are not very accurate because of the translucent character of the skin and because they affect local circulation, which directly impacts the color read.^38^^,^^39^

First, the agreement of the results after the calibration even under different illumination conditions was verified. For this, with five photographs varying the model of the cell phone and the color table, the conditions (2) and (3) were established. Then the measurements of images taken two by two were compared for each skin segment, checking the value of $\Delta E_{ab}^{*}$ between each pair.

Then the comparison for conditions (2) and (3) was done in relation to condition (1) in the same way, with the reference being the average of five skin readings. This fact is justified by the high variability of measurements on the skin, thus seeking an estimated average value. In this case, comparisons for the three skin segments are made for each image.

## 3 Results and Discussion

### 3.1 Color Correction

**Fig. 8:**
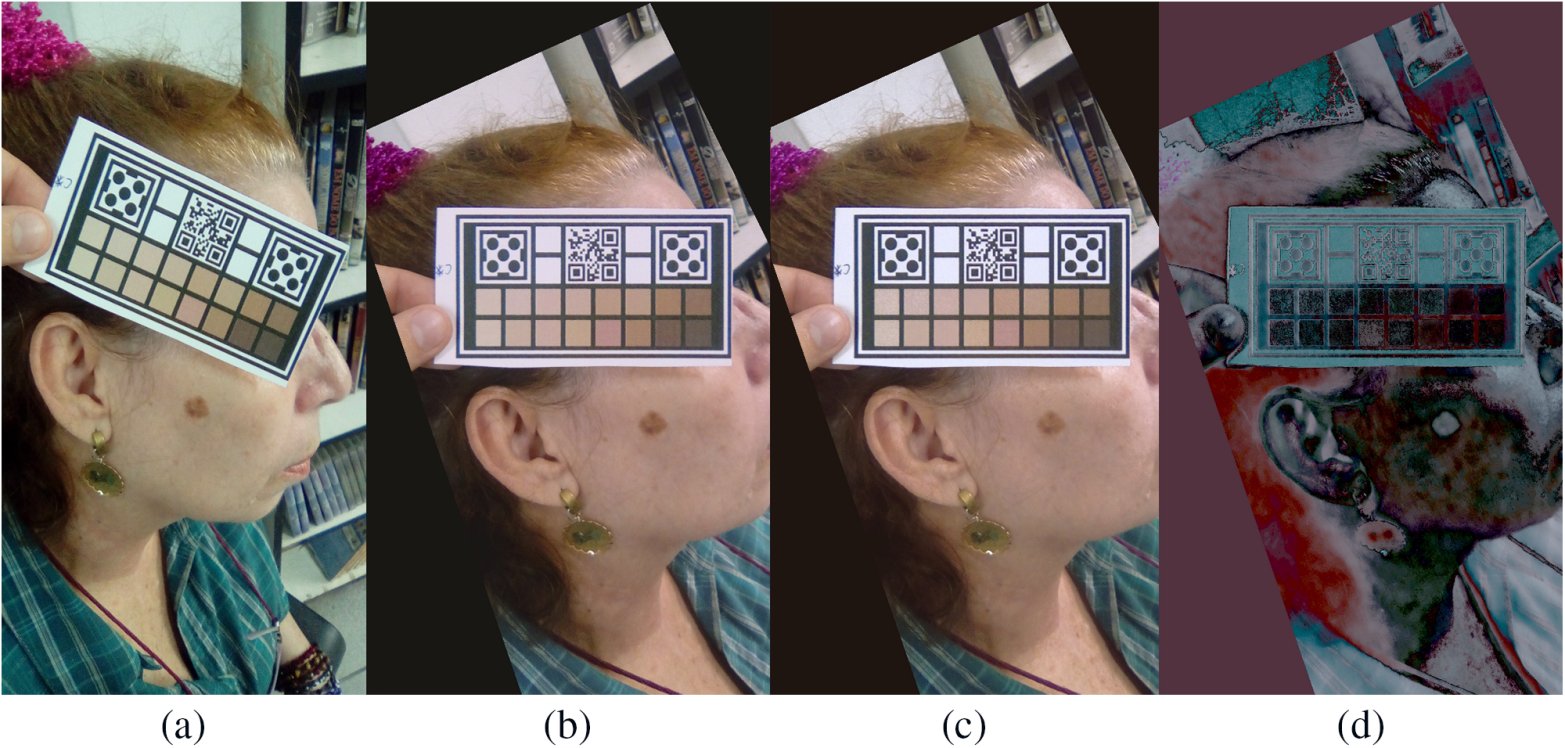
(a) Original image; (b) calibration using linear algorithm; (c) calibration using TPS-3D algorithm; and (d) logarithm of the RGB difference between calibrations.

The results comparing $\Delta E_{ab}^{*}$ when using linear and TPS-3D algorithms are shown in Table 1 and an example in Fig. 8. Values of $\Delta E_{ab}^{*}<3.00$ can be considered unnoticeable for human observers.

**Table 1:** Values comparing the results of the linear algorithm and TPS-3D to the reference.

	Linear algorithm calibration $\Delta E_{ab}^{*}$	TPS-3D algorithm calibration $\Delta E_{ab}^{*}$
Camera 1 (5 MP) and printer 1	1.0130±0.0513	0.3827±0.0405
Camera 1 (5 MP) and printer 2	1.0321±0.1957	0.3599±0.0099
Camera 2 (13 MP) and printer 1	1.6000±0.3840	0.4332±0.0632
Camera 2 (13 MP) and printer 2	1.4456±0.3308	0.5183±0.1155
Total	1.2727±0.3546	0.4235±0.0866

Also when executed on a desktop with Intel i5-4460 (3.20 GHz 4×) processor, the time difference was on average 13.3495±4.1359  s, which represents 22.28±24.18 percentual points of the time for the whole processing. The high variability in percentage is due to the resolution correction stage, which can significantly increase the total time.

As seen, the newly implemented algorithm performed better for color correction, decreasing both the final error and the standard deviation. In addition, as shown in this figure, there was little difference between some skin segments when comparing the two algorithms. Thus TPS-3D consists of a viable alternative without adding high computational complexity.

### 3.2 Illumination Correction

**Fig. 9:**
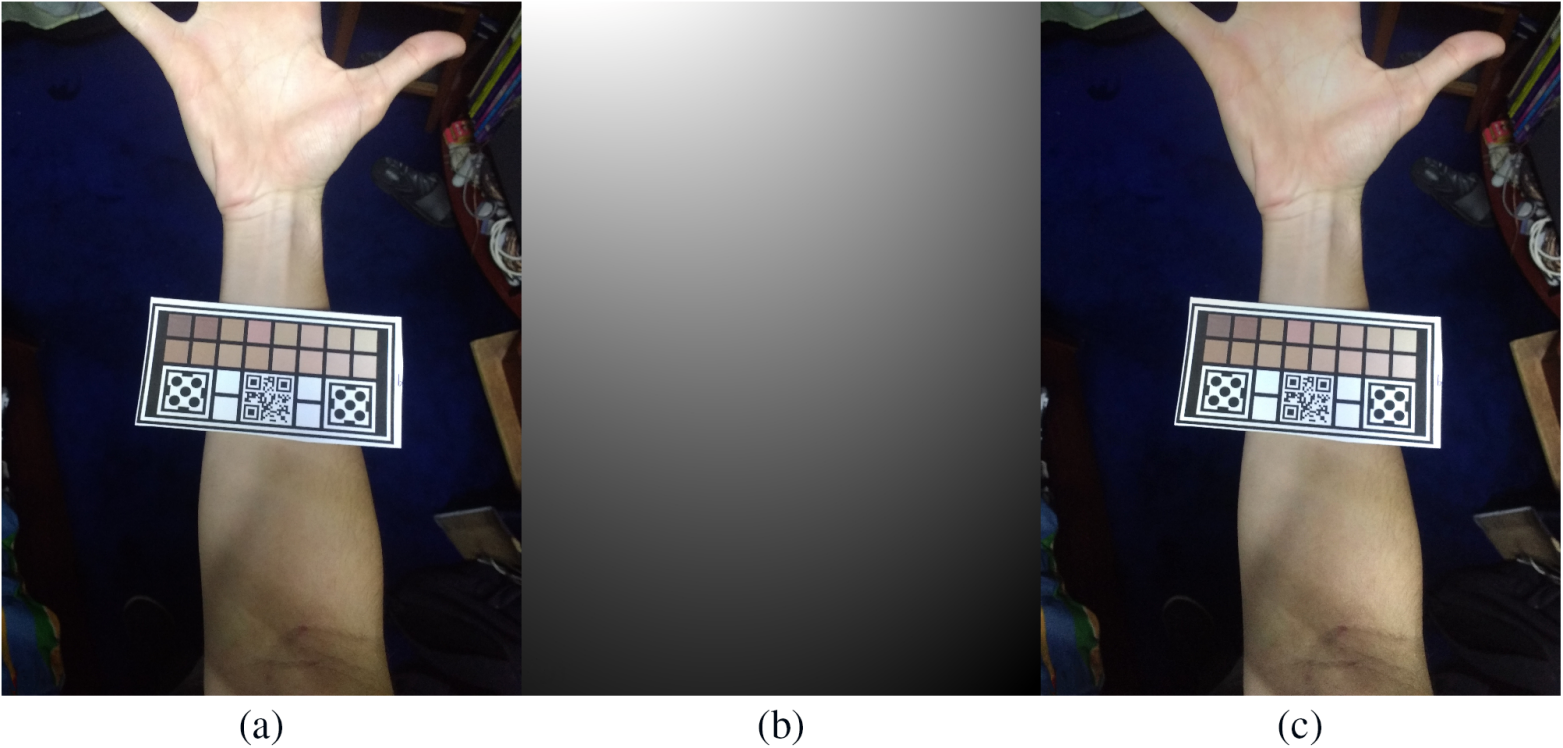
(a) Original image; (b) estimative of I(x,y) using a Gaussian filter; and (c) illumination corrected image using the illumination correction procedure.

For the illumination correction part, the same images were preprocessed using the methodology described and then were calibrated using the TPS-3D algorithm. The consolidated results are presented at Table 2 and an example of successful calibration is shown in Fig. 9.

**Table 2:** Results for $\Delta E_{ab}^{*}$ after illumination correction compared to the reference.

	$\Delta E_{ab}^{*}$ after illumination correction
Camera 1 (5 MP) and printer 1	0.4567±0.0780
Camera 1 (5 MP) and printer 2	0.3843±0.0060
Camera 2 (13 MP) and printer 1	0.3995±0.0388
Camera 2 (13 MP) and printer 2	0.3876±0.0132
Average	0.4055±0.0488

Comparing the results with those presented in Table 1, it is possible to see that the average final values were lower but not in all cases. Thus illumination correction is best used when it is checked for non-uniformity and should be placed as an option to the user when these problems are perceived.

### 3.3 Project Accessibility

**Fig. 10:**
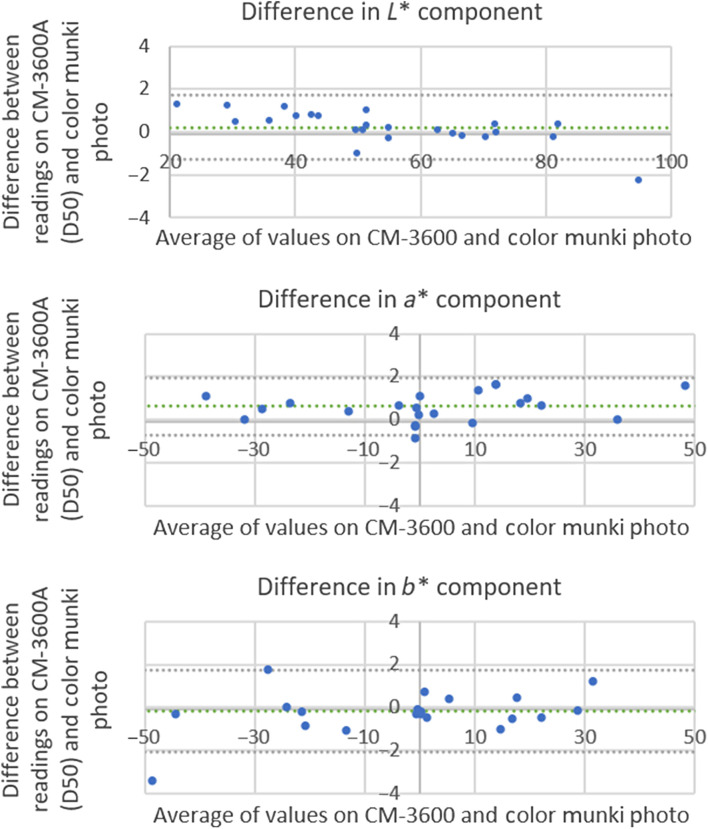
Comparison of measurements on CM-3600A converted to D50 illuminant and ColorMunki Photo.

The results for the comparison of the two devices are shown in Fig. 10 and contain the analysis for the components L*, a*, and b* of the values read for the same illuminant without calibration.

As seen, the similarity between the readings was high, obtaining an average $\Delta E_{ab}^{*}$ equivalent of 0.55±1.38, in addition to low limits of agreement. With that, it can be concluded that ColorMunki Photo represents a lower cost and greater portability equipment, without jeopardizing the measurement reliability.

### 3.4 Temporal Degradation

**Fig 11:**
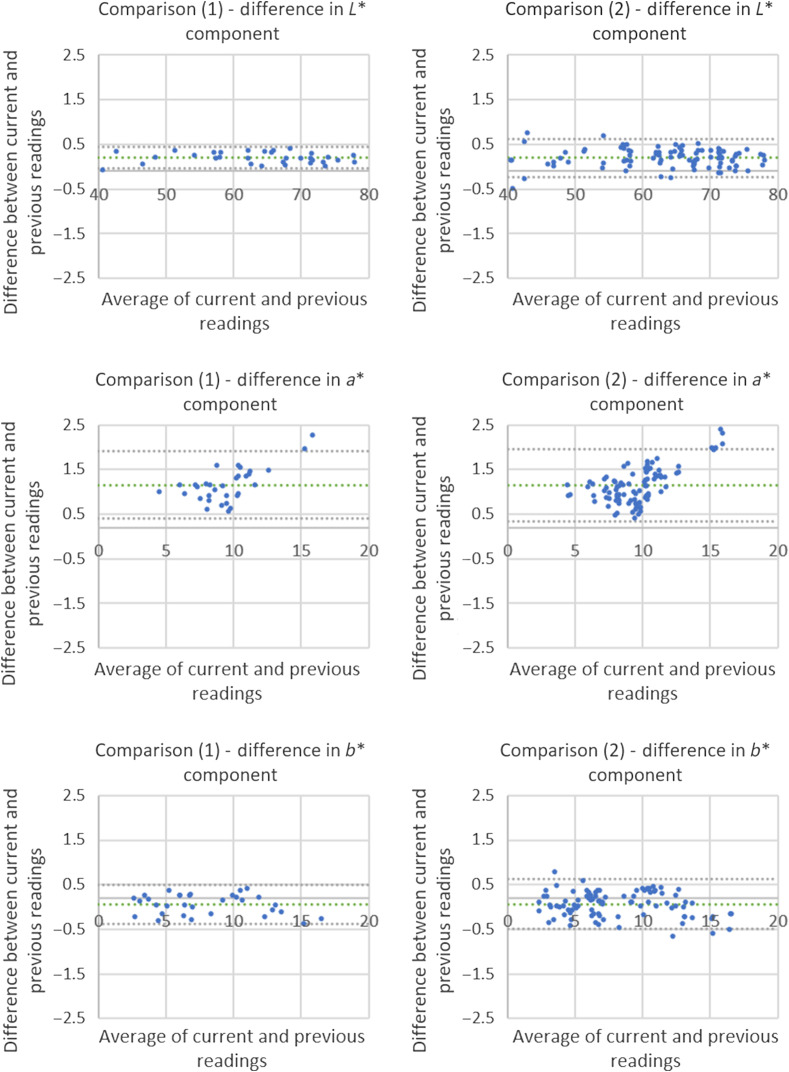
Difference for L, a*, and b* components of readings from the same device taken with an interval of 13 months.

Due to acquisition problems, only 92 out of the 96 segments were used for both proposed comparisons, whose results for each analysis are shown in Fig. 11.

It is possible to observe that, in this analysis, the variations presented for the devices in the comparison (1) were slightly high, however, when analyzed together, considering the limits of agreement, they indicate that the printed models did not present significant variation in time.

Based on the averages and standard deviations obtained, it is also seen that the two comparisons present similar results, pointing to the fact that the average of individual variations does not decrease the global variation.

Finally, analyzing the values of $\Delta E_{ab}^{*}$, in comparison (1) we obtained 1.21±0.25, whereas in comparison (2) the result was 1.34±0.25, both of which are low and reinforce the conclusions reached.

### 3.5 Skin Measurements

**Fig. 12:**
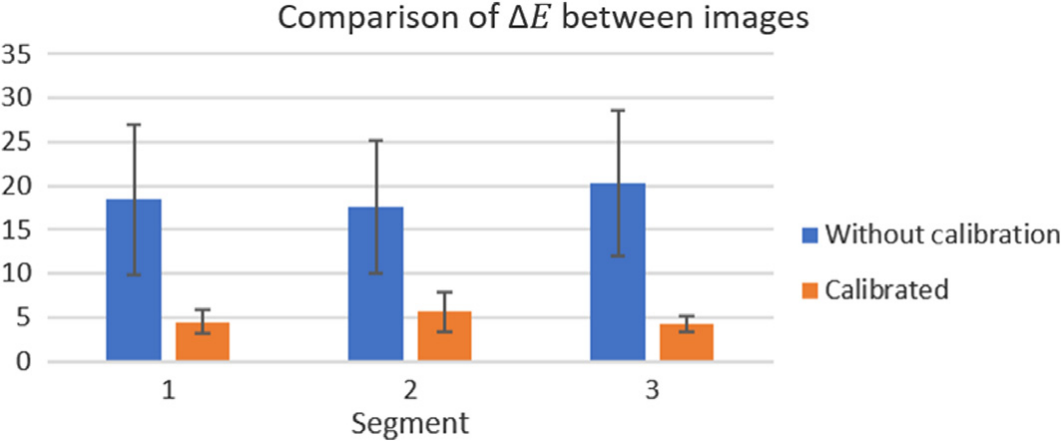
Comparison of $\Delta E_{ab}^{*}$ on segments of different images before and after calibration using the TPS-3D algorithm.

The results for the first part of this section, comparing images from the same skin segments before and after calibration, are shown in Fig. 12.

It is possible to observe that the same skin segments before calibration could have differences as high as 30 units in the metric, with an average of 18.81±4.85. After processing, this value dropped to 4.85±1.72, indicating greater similarity between the corrected colors, even when they were not part of the set used in the interpolation algorithm.

Despite this fact, the average value was still high when compared to thresholds considered for human perception ($\Delta E_{ab}^{*}<3.00$).^20^ Some possible reasons may be the presence of local shadows in the segments caused by adjacent parts or the analyzed skin tone is not covered in the color space used in the calibration. Possible future improvements include resizing the device as well as in-depth analysis of the colors to be included in the device considering distortions during the printing process.

**Fig. 13:**
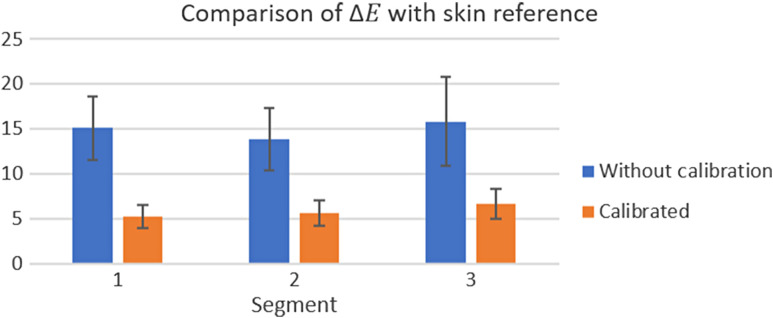
Comparison of $\Delta E_{ab}^{*}$ on segments with skin reference before and after calibration using the TPS-3D algorithm.

With the consistency of the values after the calibration, the comparison of the results in relation to the reference was done in the same way. The results are shown in Fig. 13.

In this analysis, the average dropped from 14.93±4.11 before calibration to 5.85±1.61 after the process, making the reading more reliable in relation to the objective, even though this result may be improved in the future.

## 4 Conclusion

In this paper, the calibration methodology developed shows significant improvements in the measurement of skin color compared to the procedure without calibration. This fact can be seen directly in the greater similarity of the color obtained after processing images with different cameras and illumination conditions, as well as being closer to that obtained in direct skin color reading.

It is also seen that the process can be carried out at a lower cost of implantation through reliable measurements with simpler equipment and without the observation of significant deterioration of the colors printed over time. In addition, the implemented algorithms enable greater robustness of the procedure and consistent results between executions. Therefore, a distributed calibrated device obtained from only one printer and a colorimeter or spectrophotometer can be created and used together with different phone cameras and scenarios to yield consistent results. Also only a computer is necessary to run the software, which in the future can also be used as a phone application or even a web service.

As possibilities for future improvements, the study of the colors used in the physical device can be cited so that the range of skin tones is as wide as possible considering the distortions obtained in printing. With this, there is also the possibility of measuring skin tones using non-contact equipment for results with greater fidelity.

Finally, for a good calibration process, photography with uniform illumination (or execution of the illumination correction step if variation is observed) and without shadow regions is recommended. Thus better results are found when the physical device is perpendicular to the light source. If the image obtained appears to present great color distortions, it is recommended to take a new photograph since the degrading factors mentioned may have affected the processing.

## Code, Data, and Materials Availability

The code for this project is made available at https://github.com/yargo13/correct_image. Data and materials supporting the results reported in the manuscript can be requested by contacting the corresponding author.
